# Chain splitting of insulin: an underlying mechanism of insulin resistance?

**DOI:** 10.1038/s44324-024-00042-1

**Published:** 2024-12-18

**Authors:** Christian N. Cramer, František Hubálek, Christian Lehn Brand, Hans Helleberg, Peter Kurtzhals, Jeppe Sturis

**Affiliations:** 1 Novo Nordisk A/S, Maaloev, Denmark; 2 Novo Nordisk A/S, Soeborg, Denmark

**Keywords:** Type 2 diabetes, Pre-diabetes, Metabolic diseases

## Abstract

Despite decades of intense research, the mechanisms underlying insulin resistance are still poorly understood. What if one of the major causes of insulin resistance is not related to defects in the target tissues and/or insulin receptor signaling, but rather to a reduced survival of endogenously secreted insulin on its way to activating the receptor on the cell surface of the target tissues? Here, we present data and lay out arguments in support of this novel hypothesis, which is fundamentally different from the common view that insulin resistance is caused by the body’s cells becoming less sensitive to insulin.

## Introduction

It was recently demonstrated that the major metabolic degradation pathway for the insulin analog insulin icodec in human plasma is due to the separation of the A- and B-chains of insulin by a thiol-disulphide exchange reaction (chain splitting)^1^. Furthermore, disulphide bonds in native human insulin (HI) were also shown to be subject to chain splitting when exposed to exogenous thiols in solution, and the rate of this degradation depends on redox potential, with lower redox potential leading to more chain splitting^1^. Moreover, chain splitting of HI in our redox assay occurs at redox potential values typical for human plasma^2^. In this paper, we present data demonstrating that chain splitting of HI in vivo has physiological relevance by showing that this phenomenon occurs (1) in human plasma and (2) in vivo to a substantial extent when HI is infused to rats. We present evidence from the literature supporting the novel hypothesis that redox-mediated changes in chain splitting may be a direct mechanism of insulin resistance.

## Results

**Fig. 1:**
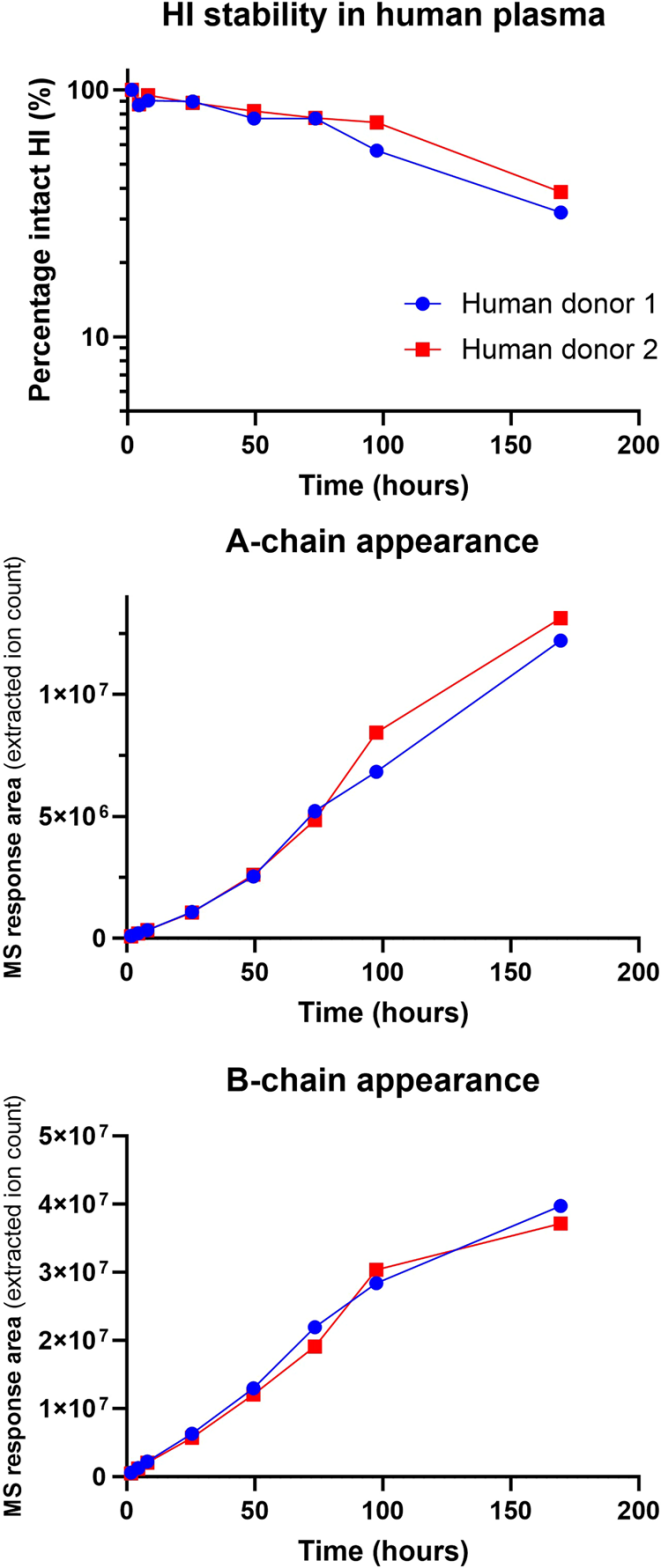
In vitro human plasma stability of HI. The disappearance of intact HI over time (top), is correspondingly associated with the appearance of the A-chain (middle) and B-chain (bottom). The exact monoisotopic masses of the observed A- and B-chains correspond to all cysteines being in the disulphide state.

**Fig. 2:**
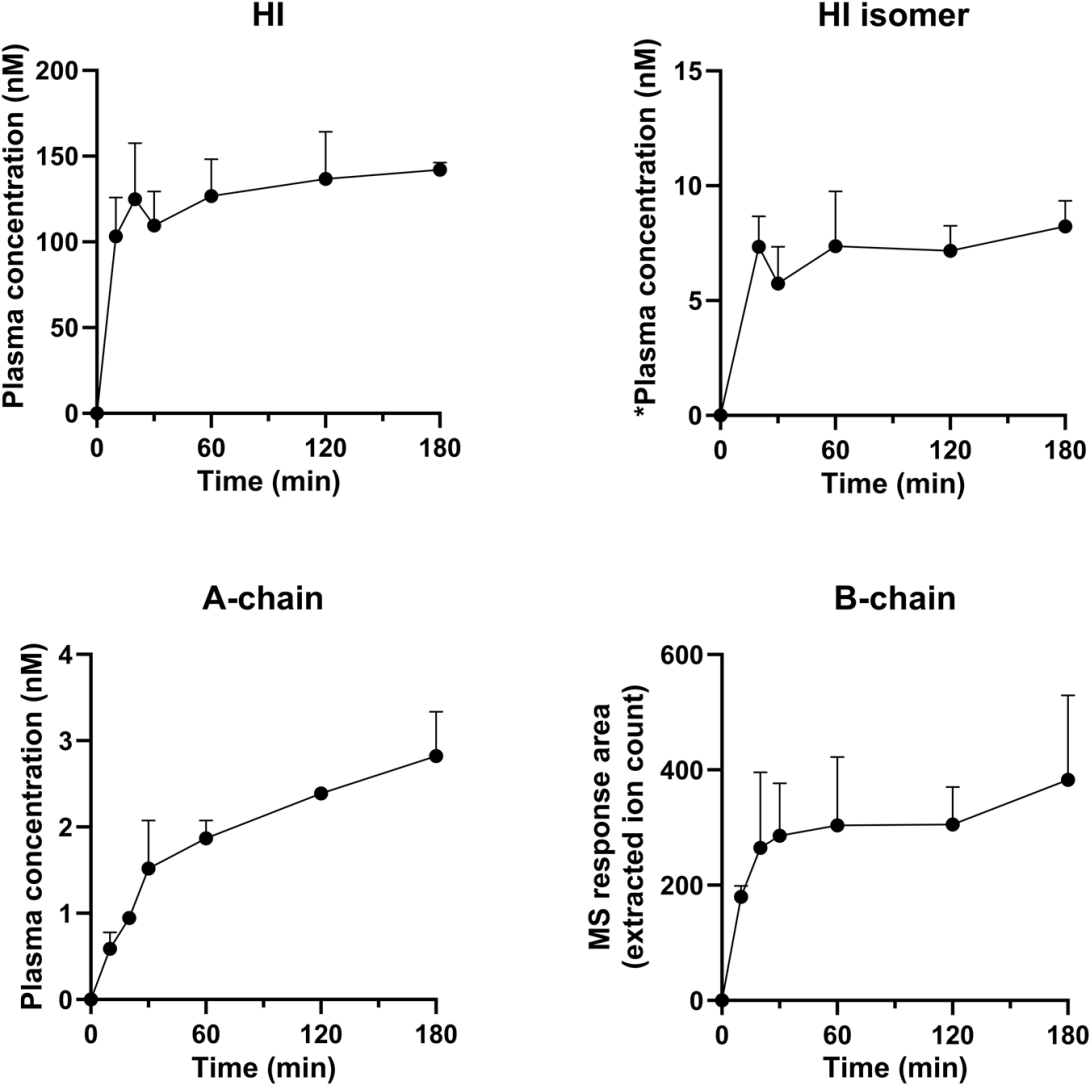
Plasma exposure (mean+SD) of HI, a HI isomer, the A- and B-chain during the rat clamp experiment. *semi-quantification of the HI isomer was done using intact HI as the reference material, assuming equal ionization efficiency of the HI isomer.

**Fig. 3:**
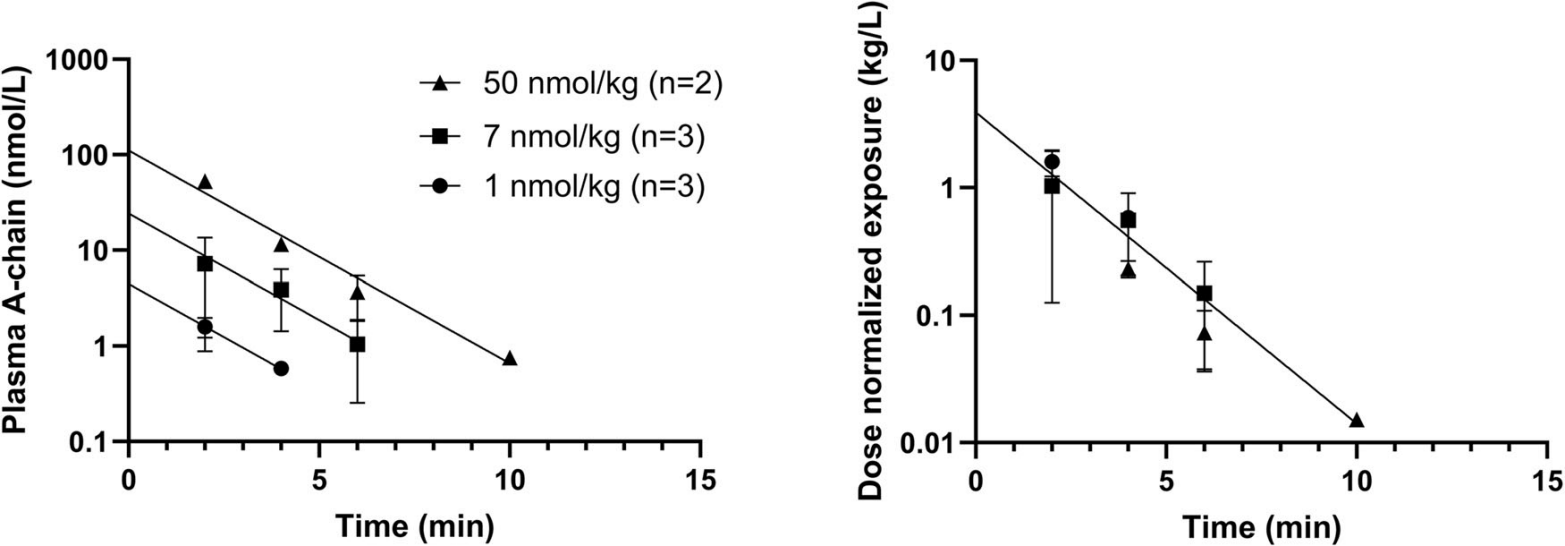
Plasma exposure (mean±SD) of A-chain following i.v. bolus administration to rat at three different doses. The left panel shows absolute concentrations, the right panel shows dose-normalized values. Vd: 0.26 L/kg, 95% CI [0.17;0.53], t½: 1.2 min, 95% CI [1.0;1.5], Cl: 0.14 L/kg/min, 95% CI [0.09;0.20].

Figure 1 shows disappearance of HI and a corresponding appearance of both insulin A- and B-chain upon incubation in human plasma. These results demonstrate that chain splitting is relevant not only for the acylated insulin analogs as published recently^1^, but also for native HI. Furthermore, in the hyperinsulinaemic euglycaemic clamp study in rats using HI, in addition to HI, we also detected A- and B-chain, as well as an isomer of HI in plasma as shown in Fig. 2. Based on the plasma A-chain levels in the clamp study and on the A-chain clearance kinetics determined in the separate PK experiment (Fig. 3), we estimate that the A-chain appearance rate (i.e., the rate of HI chain splitting) in the clamp study corresponds to 0.40 nmol/kg/min or approximately 20% of the HI infusion rate, clearly demonstrating that chain splitting is an in vivo relevant degradation mechanism also for HI.

## Discussion

We have demonstrated that a considerable proportion of HI is degraded by separation of the A- and B-chains via a disulphide bond exchange process in human plasma and when administered to rats. The disulphide exchange process for HI and bovine insulin has previously been described in detail^1,3^, including formation of free A-chain and free B-chain as well as formation of two insulin isomers^3,4^. It was also predicted that the phenomenon could occur in circulation in vivo, but only to a negligible extent^3^, in stark contrast to our observations.

Is there any literature that suggests a change in chain splitting of endogenous insulin is relevant to insulin sensitivity? The degree of chain splitting is highly dependent on the redox potential^1^, so we searched for literature describing changes in redox potential in biological settings. The glutathione redox couple, consisting of the reduced (GSH) and oxidized (GSSG) forms, plays a crucial role for the redox status in living organisms. Inhibition of cellular glutathione synthesis has been examined in a series of articles either by buthionine sulphoximine (BSO) inhibition of the rate-limiting enzyme in the glutathione synthesis, gamma-glutamylcysteine synthetase (gamma-GCS)^5^, or by inhibiting its synthesis by gene knockout of Glutamate-Cysteine Ligase Modifier Subunit (Gclm (-/-)) in mice^6–9^. In addition to lowered plasma GSH levels, inhibition of glutathione synthesis led to increased insulin sensitivity^5,7–9^ and improved glucose tolerance^8^^,9^, which was clearly unexpected. Furthermore, both GSH/GSSG and Cys/CysS plasma redox potentials were increased in the knockout mice^8^, as would be expected when the supply of GSH is greatly inhibited. These results in combination with the finding that less chain splitting occurs at higher redox potential^1^ are compatible with decreased degradation of circulating insulin via chain splitting mediated by lowered plasma GSH levels/increased plasma redox potential playing a pivotal role in the observed increased insulin sensitivity.

Literature reports on the redox state of plasma in different diseases differ. In many diseases as well as in aging, circulating low molecular weight thiols were reported to become more oxidized^10^, while other reports suggest a link between increased GSH/decreased plasma redox potential and insulin resistance and/or risk of type 2 diabetes development. For example, GSH/GSSG plasma redox potential in general was lower in obese insulin resistant than in lean insulin sensitive persons^11^. The possibility that diet-induced changes in plasma redox potential could be causative in modulating insulin resistance via altered chain splitting is intriguing since diet is known to affect both redox potential and risk of developing type 2 diabetes. For example, decreased dietary intake of sulphur amino acids increased Cys/CysS plasma redox potential, and subsequent replenishment of dietary intake of sulphur amino acids decreased Cys/CysS plasma redox potential^12^. In another publication, increased dietary intake of sulphur amino acids was associated with an increased risk of development of type 2 diabetes^13^. Increased GSH/decreased redox potential will lead to increased rate of insulin chain splitting according to our results, and increased insulin secretion will be required to compensate for such increased insulin degradation.

**Fig. 4:**
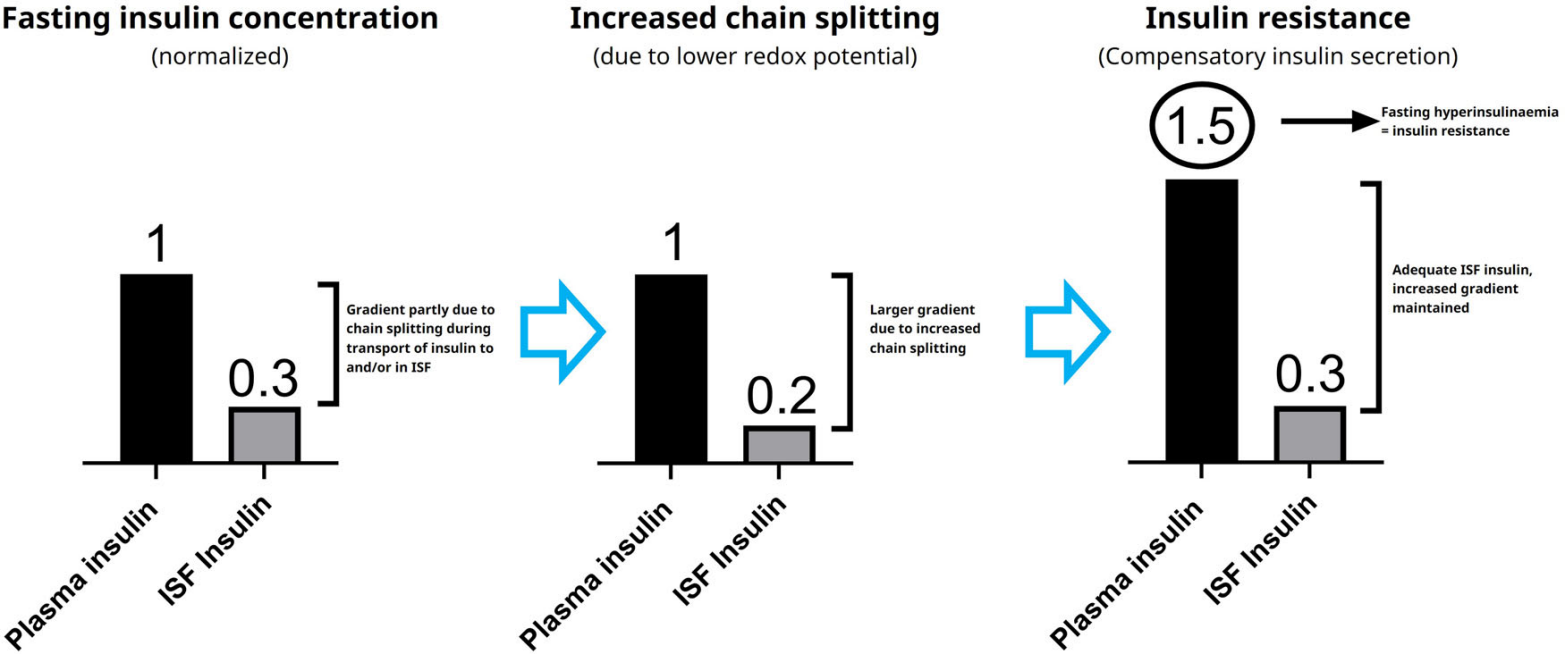
Cartoon illustrating how increased insulin chain splitting during transport to and/or in ISF will result in increased plasma insulin/insulin resistance. The left panel represents the insulin concentration gradient from published data^18^ in healthy individuals. The middle panel illustrates how increased chain splitting will result in a larger gradient according to our hypothesis, leading to compensatory insulin secretion, plasma hyperinsulinaemia and thus insulin resistance as shown in the right panel.

Understanding the influence of thiol redox shifts in plasma is complex since the various thiol-disulphide pairs are not at equilibrium^14^. Additionally, cells contain a much higher concentration of low molecular weight thiols compared to plasma (for instance, cellular levels of GSH are 2–17 mM^15^, while plasma levels of GSH are 4.9–7.3 µM^16,17^), and the different thiol pairs are more reduced in the cells than in plasma. Given that insulin activates receptors on the cell surface, namely in the interstitial fluid (ISF), it is reasonable to assume that the thiol pools in this compartment might be influenced by local cellular thiol release. Consequently, the local thiol concentrations and redox status in the ISF might differ from those in plasma. Currently, there seems to be no detailed analysis available concerning redox status in ISF within existing literature. Insulin must cross the endothelium on its way from blood to ISF. The local redox status of the endothelium may also be influenced by release of thiols, and this may affect the degree of insulin chain splitting that occurs when insulin crosses the endothelial barrier. The occurrence of chain splitting during transport to and/or in ISF is compatible with the insulin gradient that exists between plasma and ISF^18^. Furthermore, it has been demonstrated that the transport of insulin across the capillary endothelium limits the time course of insulin action, and that direct administration of insulin into muscle reveals a fourfold increased muscle insulin sensitivity compared to the value based on plasma insulin concentrations^19^, which is also compatible with chain splitting during transport of insulin from plasma to ISF playing a role. In case of a relative increase in degradation rate of insulin before its activation of and internalization by the insulin receptor (whether in ISF and/or during transport from plasma), the lower ISF insulin concentration would lead to reduced muscle and adipose tissue glucose uptake and hyperglycaemia, immediately followed by compensatory insulin secretion via glucose feedback. This would result in plasma hyperinsulinaemia to compensate for the increased plasma/ISF gradient, thereby restoring glucose uptake and maintaining normoglycaemia in individuals with beta cells that are able to compensate. Such individuals would, by definition, be insulin resistant. A cartoon explaining the chain of events according to this hypothesis is shown in Fig. 4. The compensatory insulin secretion and hyperinsulinemia that would follow our hypothesis for insulin resistance fit nicely into the well-established concept of a hyperbolic relationship between insulin secretion and insulin sensitivity^20,21^. Conversely, a decreased rate of insulin chain splitting would result in a reduced gradient, a reduction in insulin secretion and plasma insulin levels while maintaining normoglycemia, as observed in the mouse experiments with inhibition of glutathione synthesis. Measurements of plasma C-peptide in those animal models were not reported but would be important to document a reduction in insulin secretion.

Additional support for our hypothesis is the observation that exercise acutely increases insulin sensitivity^22^. People with type 1 diabetes typically need to lower their insulin dose in connection with exercising due to the increase in insulin sensitivity^23^. It has been shown that Cys/CysS and GSH/GSSG ratios both decrease acutely after exercise^24^, compatible with an increase in plasma redox potential after exercise. The possibility that a decrease in insulin chain splitting contributes to the increased insulin sensitivity observed following exercise should be evaluated.

Also, there is diurnal variation in plasma redox status^25^ as well as diurnal variations in glucose metabolism, insulin sensitivity, plasma insulin, insulin secretion and insulin clearance^26–28^. Disruptions of the body’s circadian clocks are known to influence insulin sensitivity and glucose metabolism^29^, and the possibility that redox mediated changes in insulin chain splitting are directly involved in this complex interplay is intriguing and deserves consideration.

Considering that as much as 80% of insulin secreted by the pancreas is cleared by the liver during the first pass^30^, the possibility that part of this clearance is due to chain splitting should be considered. Since the liver contributes greatly to the plasma’s supply of glutathione^31^, it stands to reason that the concentration of glutathione would be substantially elevated in the blood as it circulates through the liver, compared to its general concentration in plasma. It might then be conjectured that a considerable portion of the insulin that is metabolized by the liver may be attributed to chain splitting, and it is possible that changes in insulin chain splitting in the liver contribute to changes in hepatic insulin clearance. The exact contribution of different compartments in the body to chain splitting is difficult to measure but may be important. With the general understanding that GSH is beneficial for health, it is clear that the association between redox biology and insulin resistance is still incompletely understood.

In summary, we have demonstrated that HI is degraded via chain splitting upon incubation in human plasma and that the proportion of HI undergoing chain splitting in vivo is substantial when infused intravenously to rats. Therefore, chain splitting of insulin is relevant for insulin action since a change in the degree of chain splitting in circulation will alter the supply of insulin available for insulin receptor activation. Based on our results and the referenced literature, we propose that increased chain splitting of endogenous insulin prior to insulin receptor activation—throughout the body or locally—is a direct mechanism of in vivo insulin resistance. Although the causes of insulin resistance are multifactorial, concomitant tissue insulin resistance is neither a prerequisite for our proposed mechanism, nor is it excluded, and the relative role of insulin chain splitting vs cellular insulin resistance is unknown. Further studies into the role of insulin chain splitting in physiology and pathophysiology of disorders involving insulin, and research into possible new pharmacological interventions are warranted.

## Methods

### Production of HI A-chain

HI A-chain was prepared by chain splitting of HI. HI (final concentration 150 µM) was incubated with GSH (1 mM) and GSSG (0.04 mM) for 16 h at 40 °C in 100 mM phosphate buffer, pH = 8. The resulting A-chain was purified using a Jupiter C4 RP-HPLC column (5 µ, 300 Å, 250 × 10 mm, Phenomenex, Torrance, CA, USA) with a linear gradient of acetonitrile in 0.1% TFA at a flow rate of 4 ml/min delivered by Gilson purification system (Middleton, WI, USA) and freeze dried.

### In vitro plasma stability and quantification of in vivo samples by liquid chromatography with MS (LC-MS)

Human plasma was obtained from two healthy volunteers under an informed consent. The study and the informed consent were approved by the Danish Scientific Ethical Committee (VEK journal no H-D-2007-0055, approval date 09-NOV-2022). In vitro plasma stability of HI was assessed by incubation of 1 µM HI in 80% EDTA-stabilized human plasma and 20% PBS buffer (pH 7.4) at 37 °C with shaking. At selected time points (1.75, 4.5, 8, 25.5, 49.5, 73.5, 97.5, 169.5 h) one volume of the incubation mixture was subjected to protein precipitation using three volumes of 50% methanol/50% acetonitrile, followed by centrifugation and dilution of one volume of the supernatant with one volume of water before LC-MS analysis.

For quantification of the in vivo rat samples, selected plasma standard was prepared by spiking blank rat plasma with either intact HI (range: 0.45–8300 nM) or the A-chain reference standard (range: 0.05–200 nM) consisting of 2 out of 3 isomers^1^. Prior to LC-MS analysis, the plasma standards, blank plasma and study samples were prepared by protein precipitation. For A-chain quantifications one volume of plasma sample was precipitated using three volumes of 50% methanol/50% acetonitrile, followed by centrifugation and dilution of one volume of the supernatant with four volumes of water containing 1% formic acid (FA). For quantification of intact HI and relative B-chain appearance, one volume of plasma sample was precipitated with two volumes of 20% methanol/80% acetonitrile, followed by centrifugation and dilution of one volume of the supernatant with one volume of water.

The LC-MS analysis was carried out on two different LC-MS systems: a TLX-2 TurboFlow high-performance LC system coupled to a Q Exactive Orbitrap mass spectrometer (Thermo Fisher Scientific, Bremen, Germany) for analysis of in vitro plasma stability samples and in vivo A-chain quantifications, and an Acquity I-Class LC system coupled to a Synapt G2-S MS (Waters Company, U.K.) for analysis of in vivo samples for intact HI and B-chain.

On the TLX-2 LC system, the mobile phases consisted of solvent A: Milli-Q water with 5% organic solvent (50% methanol/50% acetonitrile) and 1% FA; and solvent B: Milli-Q water with 95% organic solvent (50% methanol/50% acetonitrile) and 1% FA. For the in vivo A-chain quantification a TurboFlow Cyclone 0.5 × 1000 mm column from Thermo Fisher Scientific (Bremen, Germany) was used for online extraction before elution to the analytical column, whereas the samples from the in vitro plasma stability were loaded directly onto the analytical column. The analytical column used was a XBridge Protein BEH C4, 300 Å, 3.5 µm, 2.1 × 50 mm from Waters (Wilmslow, U.K.) controlled at 60 °C, and elution was performed using a flow rate of 400 µL/min and a linear 40% gradient of mobile phase solvent B from 40% to 80% over 2.5 min for the in vivo A-chain quantification, and for the analysis of the in vitro plasma stability samples a linear gradient of 10–90% B over 5 min was used. The Orbitrap MS was operating in positive ionization mode with a spray voltage of 4.0 kV, and a resolution of 35 K using a single ion monitoring scan (*m/z* 1187.5–1192.5) for the in vivo A-chain quantifications and a full scan (*m/z* 300–3500) mode for the in vitro plasma stability samples. The Orbitap LC-MS data were processed using the Quan Browser in the Xcalibur software from Thermo Fisher Scientific (Bremen, Germany) with a lower limit of quantification of the A-chain in the in vivo samples in the range of 0.2–0.5 nM.

On the Acquity LC system, the mobile phases consisted of solvent A: Milli-Q water with 1% FA; and solvent B: Milli-Q water with 90% acetonitrile and 1% FA. The column used was an Acquity Peptide BEH C4 column, 300 Å, 1.7 µm, 2.1 × 150 mm from Waters (Wilmslow, U.K.) controlled at 50 °C, and elution was performed using a flow rate of 400 µL/min and a linear 25% gradient of mobile phase solvent B from 20% to 45% over 30 min. The Synapt G2-S MS was operated in positive ionization mode with a spray voltage of 3.1 kV, using the Resolution mode and recorded using mass intervals of *m/z* 1155–1170 and *m/z* 850–865 with target enhancements of *m/z* 1162 and *m/z* 858 and 0.25 s scan times, for intact HI and the B-chain, respectively (the mass spectrum of the B-chain contained characteristic multiply charged ions for the B-chain structure with monoisotopic masses of *m/z* 686.1396 and 857.4118). The Synapt G2-S LC-MS data were processed using the QuanLynx application in the MassLynx software from Waters Company (Wilmslow, U.K.) with a lower limit of quantification of HI in the in vivo samples of 4.3 nM. A mass interval of m/z 500–1500 with 1 s scan time was used for collection of full scan spectrum for qualitative analysis of the rat plasma following 180 min of infusion with HI.

### In vivo experiments

Animal procedures were approved by the Danish National Animal Experiments Inspectorate and the Novo Nordisk Ethical Review Council.

In general, the rats had free access to fresh tap water and food (Altromin 1324, Brogaarden, DK) except during fasting periods, and followed a 12:12 h light-dark cycle with light on at 6 am and were allowed to acclimatize for approximately two weeks before any procedures were initiated.

### Hyperinsulinaemic euglycaemic clamp study with HI

Male Sprague Dawley rats (Janvier Labs, France) weighing ~350 g at arrival were used. Rats were instrumented with permanent catheters in the Carotid artery (sampling) and Jugular vein (insulin and glucose infusions) 9–11 days before subjected to the clamp experiment which was performed as previously described^32^. Briefly, rats were fasted overnight, and human insulin (*n* = 2) was infused i.v. at 2 nmol/kg/min (a supramaximal dose to maximize the chance of detecting free A-chain in plasma) for 180 min while plasma glucose was measured at 10 min intervals and clamped at euglycaemia by adjusting the glucose infusion rates (GIR). Blood samples were taken at 10, 20, 30, 60, 120 and 180 min for determination of insulin and free A- and B-chain concentrations.

### I.v. bolus pharmacokinetic study of the HI A-chain

Male Sprague Dawley rats were instrumented with permanent catheters and fasted overnight as described above. The A-chain (1, 7, and 50 nmol/kg) (*n* = 2–3) was administered i.v. at time zero and blood samples were taken at 2, 4, 6, 20, 60 and 120 min post dosing. Blood samples were immediately centrifuged, and plasma stored at −20 °C until assayed.

### Data analysis

Data from the PK experiment with A-chain were analyzed by nonlinear least squares regression analysis of the dose normalized exposure values from all three doses using GraphPad Prism 9.0.1. Data could be fitted to a one-phase decay and the two parameters (intercept and rate constant) for the three different doses were not significantly different, so all the data were fitted to a one-phase decay constraining the parameters to be shared for the three different doses. The rate of chain splitting was then estimated by multiplying the A-chain exposure 180 min into the clamp experiment (2.823 nmol/L) with the A-chain clearance parameter from the PK experiment (0.14 L/kg/min). This calculation assumes that all chain splitting occurs in a single compartment (where sampling was performed) and that the A-chain concentration is in steady state at the end of the clamp experiment.

## Supplementary information

Supplementary information

## Data Availability

Data is supplied within the manuscript.
