# Publisher Correction: Chain splitting of insulin: an underlying mechanism of insulin resistance?

**DOI:** 10.1038/s44324-025-00076-z

**Published:** 2025-06-20

**Authors:** Christian N. Cramer, František Hubálek, Christian Lehn Brand, Hans Helleberg, Peter Kurtzhals, Jeppe Sturis

**Affiliations:** 1 Novo Nordisk A/S, Maaloev, Denmark; 2 Novo Nordisk A/S, Soeborg, Denmark

**Keywords:** Type 2 diabetes, Pre-diabetes, Metabolic diseases

Correction to: *npj Metabolic Health and Disease* 10.1038/s44324-024-00042-1, published online 18 December 2024

In this article Ref. 7 was incorrectly given as ‘Glutamate cysteine ligase—modulatory subunit knockout mouse shows normal insulin sensitivity but reduced liver glycogen storage. *Front. Physiol*. **7**, 142 (2016)..’ and should have read ‘Lavoie, S. et al. Glutamate cysteine ligase—modulatory subunit knockout mouse shows normal insulin sensitivity but reduced liver glycogen storage. *Front. Physiol*. **7**, 142 (2016).’. The original article has been corrected.

