# Supplementary material for: Chain splitting of insulin: an underlying mechanism of insulin resistance?
Source: NPJ Metab Health Dis. 2024 Dec 18;2:38. doi: 10.1038/s44324-024-00042-1 (PMC12118748; doi:10.1038/s44324-024-00042-1)
Supplement: Supplementary file 1 — Supplementary information [file 44324_2024_42_MOESM1_ESM.docx]

Supplementary information


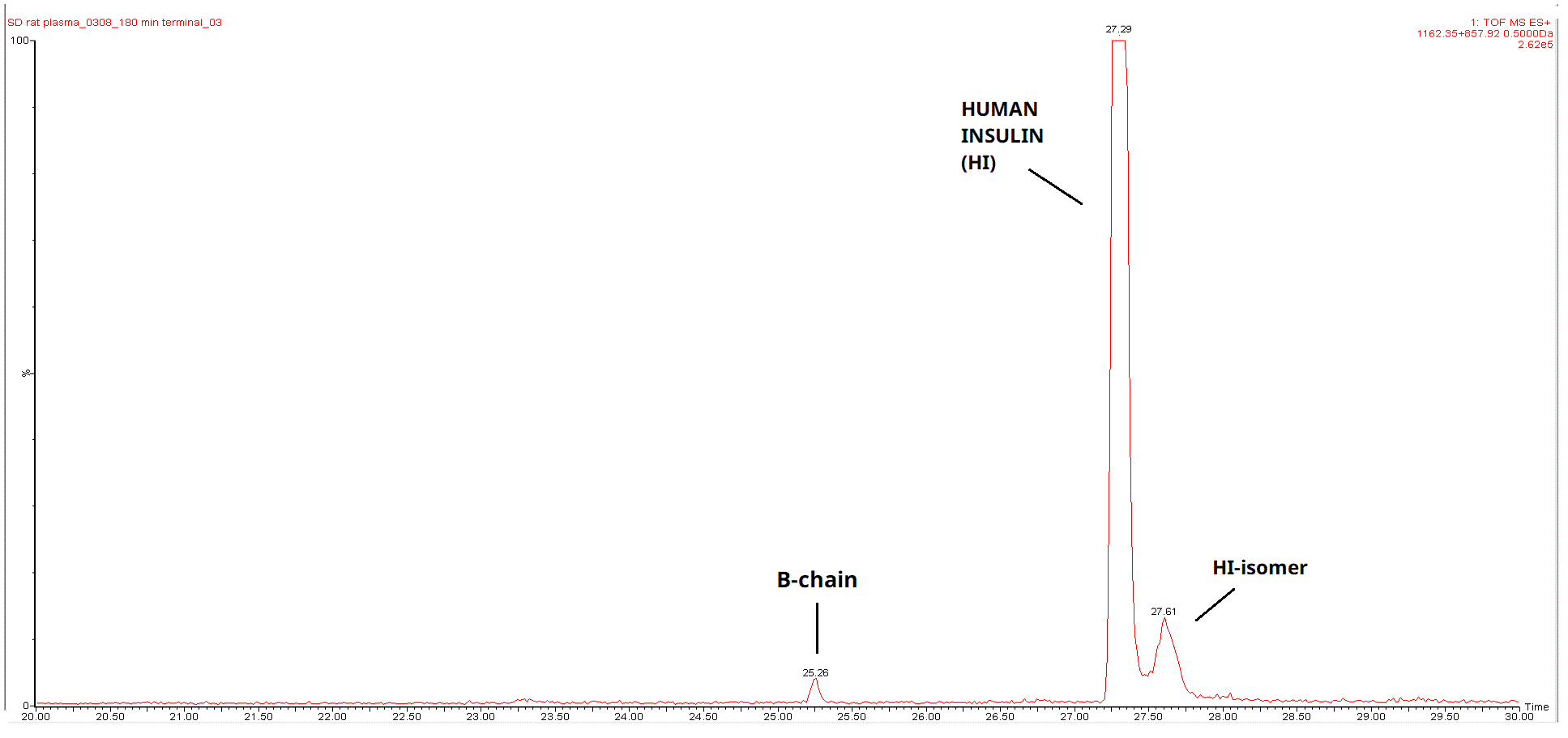


Supplementary Figure 1. LC-MS extracted ion chromatogram with peaks of HI, a HI -isomer and the B-chain in rat plasma from the clamp study


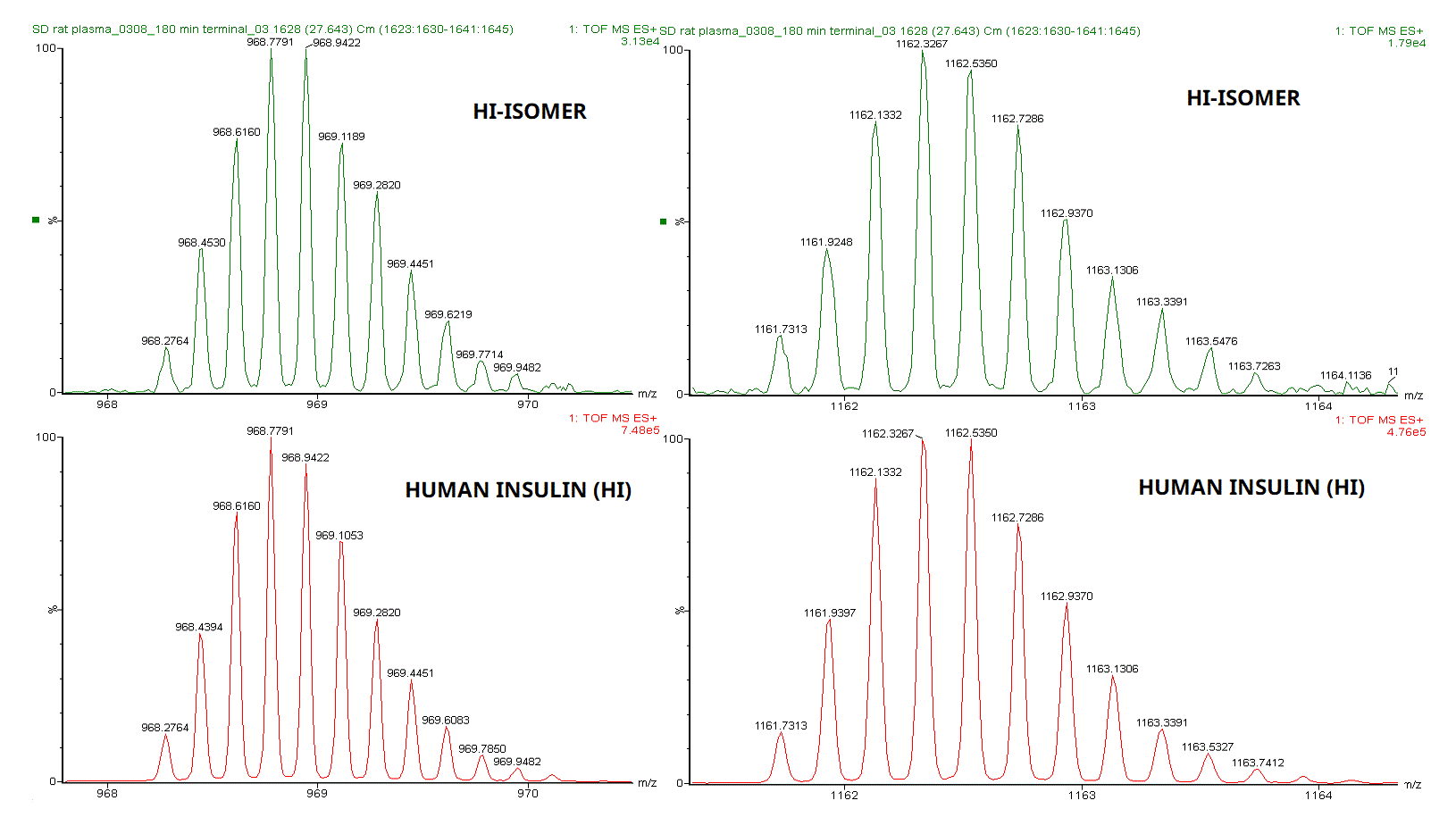


Supplementary Figure 2. Comparison of the most abundant multiply charged ion clusters of human insulin and an isomer of HI detected in plasma. [M+6H]^6+^ ions on the left- and [M+5H]^5+^ ions on the right-hand side
